# Supramolecular Polymerization of Biphenyl-Cyanostilbenes. Triggering Circularly Polarized Luminescence by Self-Assembly

**DOI:** 10.1021/acs.orglett.5c01116

**Published:** 2025-05-14

**Authors:** Miguel Fernández, Lucía López-Gandul, Rafael Gómez, Luis Sánchez

**Affiliations:** Departamento de Química Orgánica, Facultad de Ciencias Químicas, 16734 Universidad Complutense de Madrid, 28040 Madrid, Spain

## Abstract

We report on the synthesis of a series of cyano-luminogens with cyanostilbene fragments, benzamide units, and a nonplanar biphenyl core. Benzamide units drive supramolecular polymerization, forming helical *H* aggregates via kinetically controlled self-assembly. A sergeants-and-soldiers experiment revealed frustrated asymmetry amplification due to the contamination of linear dichroism. The planarization of the central π-conjugated systems enhances aggregation-induced emission affording circularly polarized luminescence-active helical aggregates.

## Graphical abstract

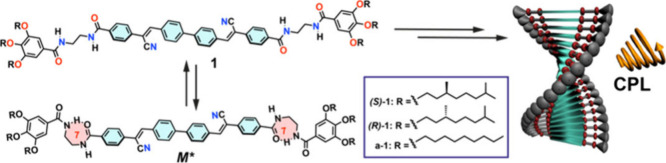

Chiral organic fluorophores represent an active field in synthetic chemistry and materials science due to the potential applications of circularly polarized luminescence (CPL) in optical technologies.^1^ The arrangement and organization of these chromophores significantly influence their chiroptical properties. Thus, the self-assembly of judiciously designed chromophores through noncovalent forces yields functional supramolecular polymers that serve as excellent models for studying these properties.^2^ Several π-conjugated systems, such as oligomers, polycyclic aromatic hydrocarbons,^3^ rylenes,^4^ allenes,^5^ and porphyrins,^6^ have been reported to self-assemble into highly organized supramolecular structures with remarkable chiroptical features. However, a common drawback of many self-assembled π-conjugated systems is the formation of *H*-type structures, where emission is suppressed due to aggregation-caused quenching (ACQ), limiting their optical applications.^7^ Nevertheless, there are some approaches to afford highly emissive and organized supramolecular ensembles. The first one is the formation of *J*-type aggregates, which exhibit a red shift in the absorption and emission maxima along with increased fluorescence intensity due to the head-to-tail arrangement within the polymeric chains.^8^ Furthermore, the generation of *H*-type aggregates showcasing an aggregation-induced emission (AIE) effect has also been reported to yield highly emissive structures. In these systems, either restricted intramolecular rotations or an effective planarization of the π-system, both increasing the conjugation efficiency, enhance the emission properties upon supramolecular polymerization.^7^ In addition, if enantiomerically enriched, these helical emissive ensembles offer a promising approach for developing new CPL active materials. While some polymers, liquid crystals, and a few small molecules have been reported as CPL emitters, materials exhibiting AIE,^9,10^ which achieve a predictable and ordered supramolecular organization of chromophores, remain unexplored. However, the supramolecular polymerization of tailored chiral monomeric units provides an effective strategy to control the arrangement of emissive scaffolds, affording efficient CPL emitters with tunable properties from the molecular level.^11–16^ A successful strategy to achieve supramolecular polymers acting as CPL emitters is the decoration of different scaffolds with cyanostilbene moieties.^17–25^ These fragments are out of the planarity in the molecularly dissolved state but experience a conformational planarization upon self-assembly, improving the corresponding fluorochromic behavior.^21^ Despite the impact of this structural motif on the CPL behavior of supramolecular polymers, the establishment of clear structure–function rules is a must.

We report herein the synthesis of a series of cyano-luminogens featuring two cyanostilbene fragments functionalized with benzamide units and peripheral chiral or achiral side chains, all anchored to a nonplanar biphenyl central core (compounds **1** in Figure 1). The benzamide units promote the supramolecular polymerization of **1**, leading to the formation of helical aggregates by a kinetically influenced self-assembly process. Additionally, we investigated the frustrated amplification of asymmetry through a kinetically influenced *sergeants-and-soldiers (SaS)* ^2^ experiment, mixing both chiral ***(S)-*** **1** and achiral **a-1**. The self-assembly of achiral **a-1** results in the formation of long and thick fibers, inducing a pronounced linear dichroism (LD) effect that pollutes the circular dichroism (CD) response. In this process, the planarization of both the cyanostilbene and biphenyl moieties enhances the emissive behavior due to the AIE effect. Furthermore, the self-assembly of ***(S)-*** **1** and ***(R)-*** **1** leads to highly emissive, helical aggregates that exhibit circularly polarized luminescence (CPL) with remarkable luminescence dissymmetry factors (*g*_*lum*_) of ∼ 0.009 (Figure 1). These findings highlight the critical role of structural design in modulating the chiroptical properties of supramolecular assemblies.

**Figure 1:**
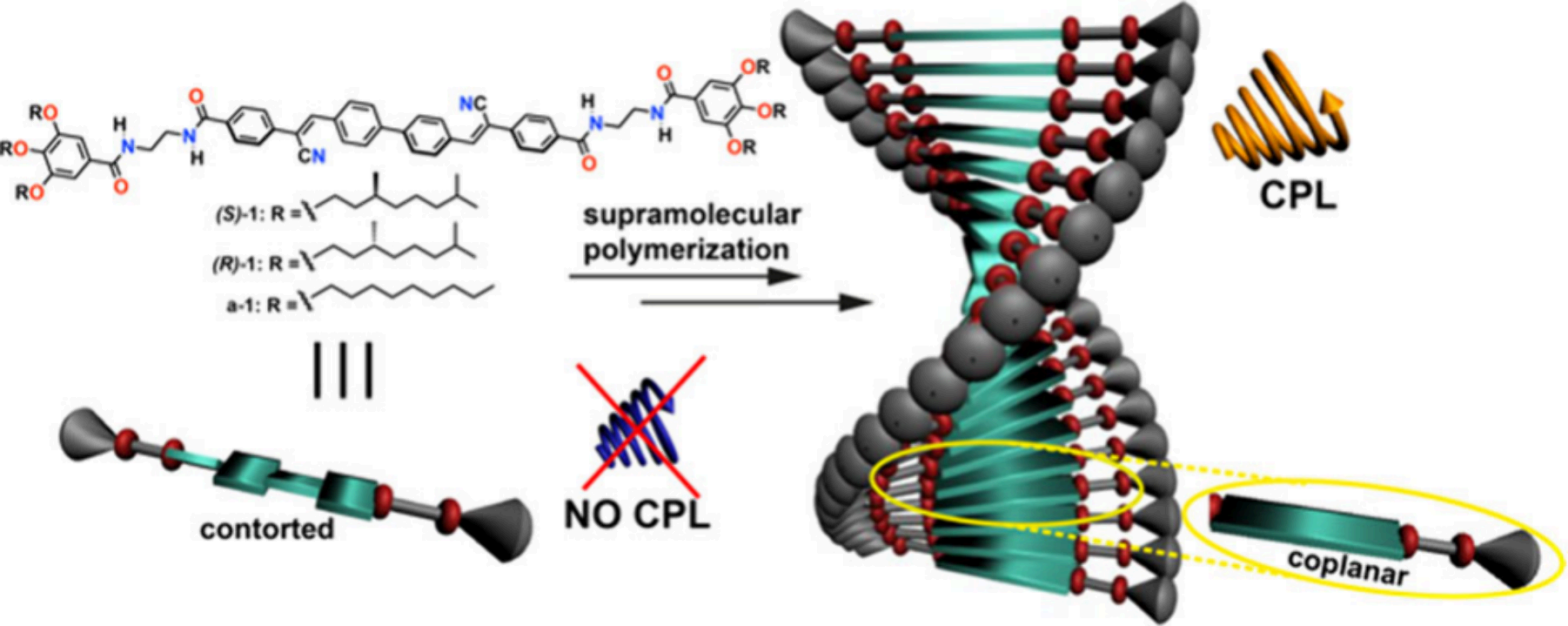
Chemical structure of the investigated cyanostilbenes **1** and schematic illustration of the helical, CPL-active supramolecular polymers formed upon self-assembly in MCH.

Luminogens **1** were straightforwardly prepared by reacting the commercially available [1,1′-biphenyl]-4,4′-dicarbaldehyde and the corresponding 4-(cyanomethyl)-benzamides **4**, previously reported by our research group.^17,24^ The addition of cold MeOH over the reaction mixture, which results from the Knoevenagel-type reaction between these two building blocks, promotes the precipitation of compounds **1**, that are collected by filtration and dried (Scheme 1). To avoid photoisomerization and/or [2 + 2] photocyclization, the sample is protected from light.^25^

**Scheme 1:**
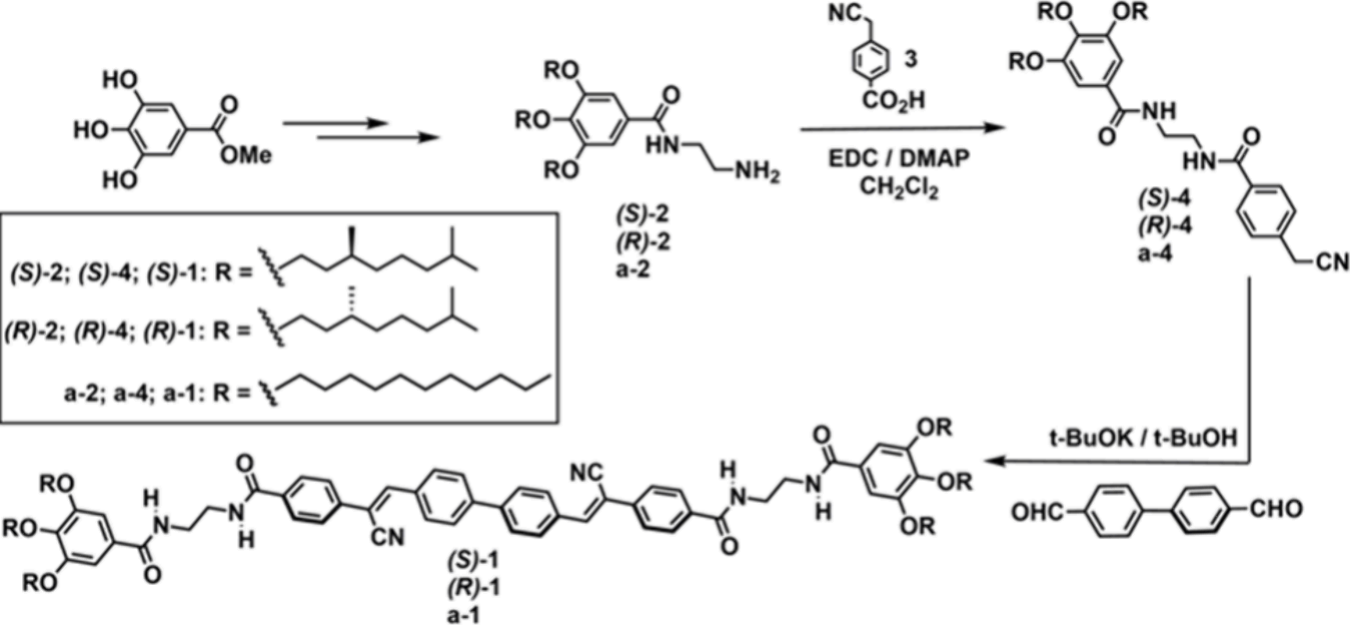
Synthesis of the Cyanostilbenes **1**

To investigate the supramolecular polymerization of the cyanostilbenes **1** and the noncovalent forces operating in their self-assembly, we have initially registered ^1^H NMR spectra in CHCl_3_ at different concentrations. Despite the ability of this solvent to solvate the investigated compounds, these experiments show the upfield shift of all of the aromatic resonances and the deshielding of the amide protons upon increasing the concentration. These findings demonstrate that the self-assembly of these luminogens is driven by the π-stacking of the aromatic backbones and the formation of an array of H-bonding interactions between the amide protons (Figures S1–S3).^17,24,25^ A further corroboration of the formation of the H-bonds between the amide functional groups stems from the FTIR spectra in a poor solvent such as methylcyclohexane (MCH), which favors the self-assembly of the investigated compounds. In this case, the NH and Amide I stretching bands appear at ∼ 3310 and ∼ 1635 cm^–1^, respectively. These values are ascribed to intermolecularly H-bonded amides (Figures 2a and S2).^17,26^ On the contrary, two NH stretching bands at ∼ 3450 and ∼ 3340 cm^–1^ are observed in the good solvent CHCl_3_. The former is attributed to the formation of intramolecularly H-bonded pseudocycles (M* in Figure S2a) by the intramolecular interaction between the NH of the inner benzamide group and the carbonyl of the outer benzamide unit (Figures 2a and S2b,c).^17,26^ The latter is ascribed to the free amides, which is further corroborated by the wavenumber of the Amide I stretching band (∼1650 cm^–1^).^17,26^ The formation of the M* species has been confirmed by registering variable-temperature (VT) ^1^H NMR spectra in CDCl_3_ at diluted conditions (total concentration *c*_*T*_ = 1 mM). These experiments show the upfield shift of the resonances ascribable to the amide protons upon increasing the temperature due to the disruption of the intramolecular H-bonds. However, unlike that observed in the concentration-dependent ^1^H NMR spectra, the aromatic resonances remain unaltered upon increasing the temperature, diagnostic of the presence of only monomeric species in solution (Figure S3).^17,26^

**Figure 2:**
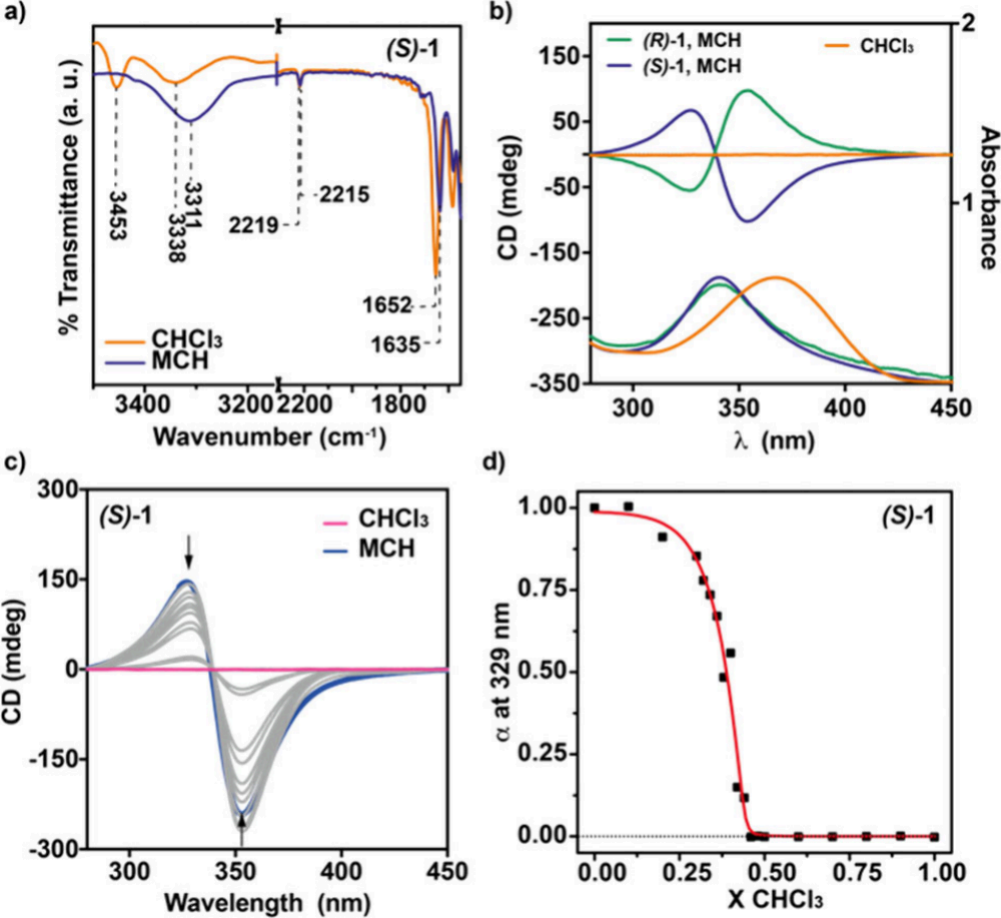
(a) Partial FTIR spectra of ***(S)*** **-1** in MCH and CHCl_3_. The dotted lines show the wavenumber values for the NH and Amide I stretching bands. (b) CD (upper part) and UV–vis spectra of ***(S)*** **-1** and ***(R)*** **-1** in MCH and CHCl_3_. (c) CD spectra of ***(S)*** **-1** in MCH, CHCl_3_, and different MCH/CHCl_3_ mixtures. Arrows in panel (c) show the changes in the dichroic response upon increasing the amount of the good solvent CHCl_3_. (d) Variation of the degree of aggregation α at λ = 329 nm upon addition of increasing amounts of a solution of ***(S)*** **-1** in CHCl_3_. The red line in panel (d) corresponds to the fit to the SD model. Experimental conditions for panels (b-d): *c*_*T*_ = 10 μM; 20 °C.

The optical properties of compounds **1** have been first explored by using VT-UV–vis. The absorption spectra of all of the studied cyanoluminogens at 20 °C and at *c*_*T*_ = 10 μM present a broad band at λ = 338 nm, assigned to the π–π* transitions of the cyanostilbene moieties.^20^ This broad band experiences a red-shift by using CHCl_3_ as the solvent (Figures 2b and S4). The bathochromic shift observed for compound **1** when using the poor solvent MCH is indicative of the formation of *H*-type aggregated species in which the aromatic moieties are arranged in a cofacial manner. The diluted solution of ***(S)*** **-1** in MCH displays a clear -/+ Cotton effect, with maxima at λ = 352 and 325 nm and a zero-crossing point at λ = 338 nm, diagnostic of an efficient transfer of asymmetry from the peripheral stereogenic centers to the central aromatic core, giving rise to *M-*type helical supramolecular polymers (Figures 2b and S5a and Table S1). As expected, the dichroic response of ***(R)*** **-1** in MCH is a mirror image of that observed for its enantiomer, thus confirming the formation of *P*-type helical supramolecular polymers (Figure 2b and Table S1).

Interestingly, aging this solution for 24 h provokes a strong increase in the intensity of the dichroic response without changing the pattern (Figure S5a). The increasing intensity of the dichroic signal upon aging of the solution demonstrates that the self-assembly of cyanostilbenes **1** is kinetically controlled by the formation of the above-mentioned pseudocycles M*. This kinetic effect has also been demonstrated by registering the cooling and heating curves of a diluted solution of ***(S)*** **-1** in MCH. Cooling down a 10 μM solution of ***(S)*** **-1** in MCH from 90 to 10 °C, applying a cooling rate of 1 °C/min, results in an uncomplete self-assembly with a weak, recovered dichroic response (Figure S5b). However, aging this sample for 24 h and applying a heating rate of 1 °C/min yields a clear nonsigmoidal curve, diagnostic of a cooperative mechanism (Figure S5b). However, and unlike previous results reported for related luminogens, no additional aggregated species have been detected.^17^ Since ***(S)-*** **1** and ***(R)-*** **1** are enantiomers, these results are assumed identical for the latter.

To quantify the stability of the supramolecular polymers formed by these luminogens, we utilized the solvent denaturation (SD) model for chiral compound ***(S)-*** **1**. In this experiment, a 24 h aged solution (therefore in the thermodynamically stable state) of the investigated scaffold in MCH, which favors self-assembly, and a solution in CHCl_3_, which favors the molecularly dissolved state due to solvation, are mixed together at different ratios but keeping constant the total concentration (see Supporting Information).^27^ The increasing molar fraction of CHCl_3_ results in clear depletion of the dichroic signal (Figure 2c). The variation of the degree of aggregation α calculated from the variation of the dichroic signal at λ = 329 nm presents a clear nonsigmoidal shape, characteristic of a cooperative supramolecular polymerization (Figure 2d).^28,29^ The thermodynamic parameters associated with the supramolecular polymerization of chiral ***(S)-*** **1** have been derived by fitting this nonsigmoidal curve. The free Gibbs energy released is 42.8 ± 2 kJ/mol, and the degree of cooperativity σ is 1.5 × 10^–3^. Furthermore, we have also visualized the morphology of the aggregated species formed by chiral ***(S)-*** **1** and achiral **a-1** by using atomic force microscopy (AFM) imaging and highly oriented pyrolytic graphite (HOPG) as the surface. The AFM images of the supramolecular polymers formed by ***(S)*** **-1** show the formation of thin and isolated fibers of 3.5 nm height (Figures 3a,b and S6). In contrast, the AFM images of achiral **a-1** show long and thick fibrillar structures of ∼ 10 nm height, most probably due to the intertwining of thinner filaments through the linear side chains (Figures 3c,d and S7).

**Figure 3:**
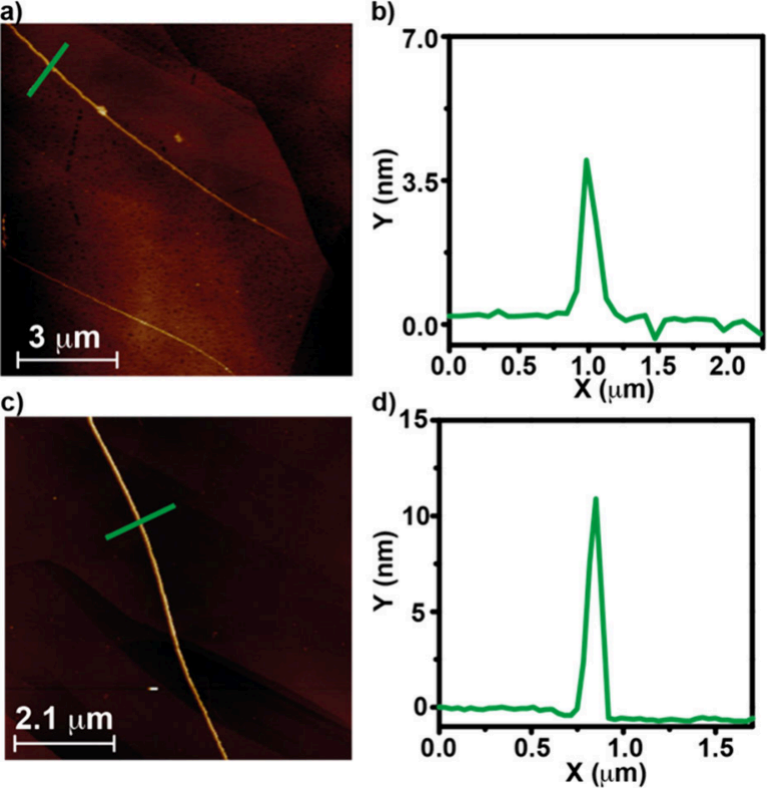
AFM images (a, c) and height profiles (b, d) of the fibrillar aggregates formed by ***(S)*** **-1** (a, b) and **a-1** (c, d) onto HOPG as the surface. The height profiles correspond to the green lines in panels (a) and (c). Experimental conditions: MCH, *c*_*T*_ = 10 μM; 20 °C.

The formation of the M* species reveals that the supramolecular polymerization of the investigated luminogens is kinetically controlled. However, we have investigated the potential amplification of asymmetry by performing a *SaS* experiment, in which two solutions of chiral ***(S)*** **-1**, acting as sergeant, and achiral **a-1**, acting as soldier, in MCH and *c*_*T*_ = 10 μM are mixed together changing the ratio of the chiral sergeant but keeping constant the total concentration.^25^ This experiment showed no amplification of asymmetry, since a linear increase in the dichroic response is observed upon adding increasing amounts of the chiral sergeant ***(S)*** **-1** (Figure S8a). To our surprise, the *SaS* experiment also showed that pristine, cyanostilbene **a-1** presents a monosignated dichroic response despite lacking any element of asymmetry in its structure. This dichroic response has been demonstrated to be caused by a remarkable LD, most likely due to the formation of long and thick fibers, as shown in the corresponding AFM images (Figure S8b).^30^

Finally, we investigated the emissive properties of chiral cyanostilbenes ***(S)-*** **1** and ***(R)-*** **1**. Previous UV–vis studies indicate the formation of *H*-type aggregated species, which often exhibit an ACQ effect in their emissive behavior (Figure 2b). In their molecularly dissolved states, both chiral compounds display a broad blue emission with a maximum at λ = 432 nm (Figure 4a). The supramolecular polymerization of ***(S)-*** **1** and ***(R)-*** **1** induces a pronounced bathochromic shift in the emission maximum (λ = 486 nm), accompanied by a remarkable 8-fold increase in emission intensity (Figure 4a). This enhancement is visible to the naked eye when solutions of these chiral scaffolds in MCH/CHCl_3_ mixtures at varying ratios are irradiated with light (Figure 4b). The observed AIE effect can be attributed to the planarization of both the contorted cyanostilbene and biphenyl moieties present in the chemical structure of compounds **1**.^17–25^ Additionally, the formation of helical aggregates exhibiting an AIE could lead to CPL activity. To investigate this, we measured CPL in both the monomeric and aggregated states. As expected, while the monomeric species exhibit no CPL, supramolecular polymerization of ***(S)-*** **1** and ***(R)-*** **1** results in intense CPL spectra with opposite signs, centered at λ = 474 nm, and remarkable *g*_lum_ values of ∼ 0.009 (Figure 4a and Table S1). These results confirm that the CPL activity of the supramolecular polymers follows helicity dictated by the stereogenic centers in the peripheral side chains.

**Figure 4:**
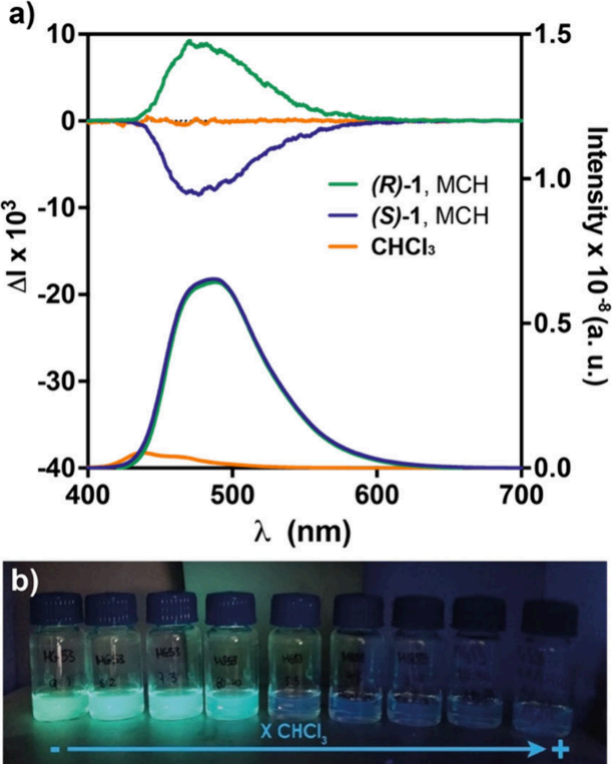
Photoluminescence and CPL spectra of ***(S)-*** **1** and ***(R)-*** **1** in its monomeric (CHCl_3_, *c*_*T*_ = 10 μM) and aggregated (MCH, *c*_*T*_ = 10 μM; 20 °C) states (λ_exc_ = 350 or 374 in CHCl_3_ and MCH, respectively). (b) Picture of 10 μM solutions of ***(S)*** **-1** in mixtures of MCH and CHCl_3_ at different ratios upon irradiation at λ = 364 nm.

In conclusion, we present the synthesis of a series of cyano-luminogens incorporating two cyanostilbene fragments functionalized with benzamide units and peripheral chiral or achiral side chains, all attached to a nonplanar biphenyl central core. The benzamide units facilitate the supramolecular polymerization of compound **1**, resulting in helical aggregates through a kinetically controlled self-assembly. Additionally, we explored the frustrated amplification of asymmetry via a *SaS* experiment, combining chiral ***(S)-*** **1** and achiral **(a)-1**. The self-assembly of achiral **a-1** produces long, thick fibers that generate a strong LD effect, which interferes with the CD response. The planarization of the cyanostilbene and biphenyl moieties enhances aggregation-induced emission (AIE). Furthermore, the self-assembly of chiral ***(S)-*** **1** and ***(R)-*** **1** results in highly emissive, helical aggregates that exhibit CPL activity with remarkable *g*_*lum*_ values. These findings underscore the crucial role of structural design in tuning the chiroptical properties of supramolecular assemblies.

## Supplementary Material



## Data Availability

The data underlying this study are available in the published article and its Supporting Information

## Notes

Experimental procedures, characterization data, additional figures and NMR spectra for all new compounds (PDF)
