# Supplementary material for: Supramolecular Polymerization of Biphenyl-Cyanostilbenes. Triggering Circularly Polarized Luminescence by Self-Assembly
Source: Org Lett. 2025 May 14;27(25):6561–5. doi: 10.1021/acs.orglett.5c01116 (PMC12210250; doi:10.1021/acs.orglett.5c01116)
Supplement: Supplementary file 1 [file ol5c01116_si_001.pdf]

Supramolecular polymerization of biphenyl-cyanostilbenes. Triggering circularly polarized luminescence by self-assembly.

Miguel Fernández, Lucía López-Gandul, Rafael Gómez, and Luis Sánchez*

Departamento de Química Orgánica I, Facultad de Ciencias Químicas, Universidad Complutense de Madrid, 28040 Madrid, Spain; e-mail: lusamar@quim.ucm.es

Table of Contents

1.- Supplementary Figures S-2

Concentration-dependent ^{1}H NMR spectra of compounds **1** S-2

FTIR spectra of compounds ***(R)-1*** *and* ***a-1*** S-3

VT-^{1}H NMR spectra of compounds **1** S-4

UV-Vis spectra of ***a-1*** S-5

Time dependent CD spectra and cooling/heating curves of ***(S)-1*** S-5

g_{abs} and g_{lum} values S-5

AFM images S-5

SaS experiment S-6

3. Experimental section S-7

4. Synthetic details and characterization S-8

5. Collection of NMR spectra S-11

1. Supplementary Figures

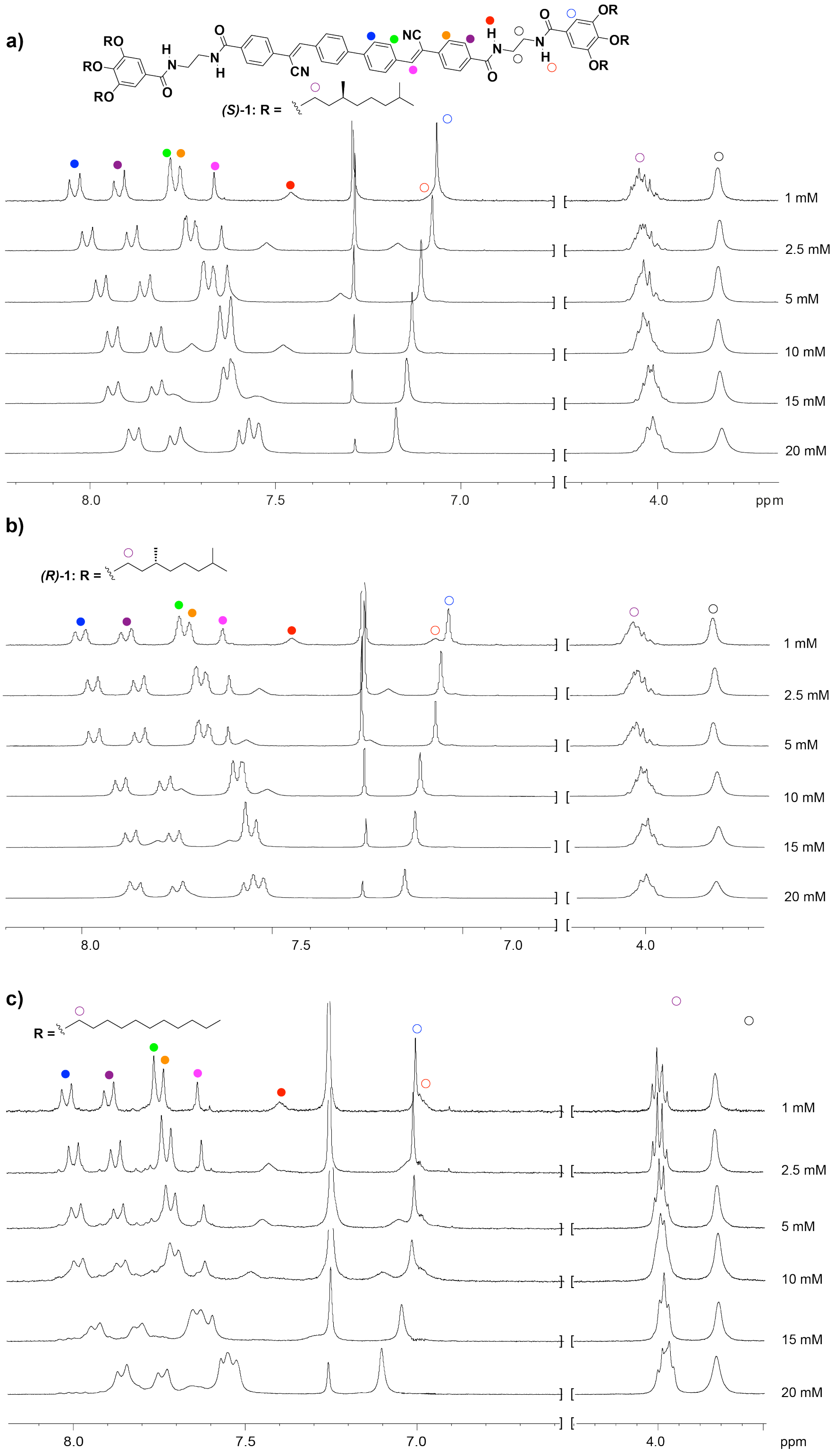

Figure S1. Concentration-dependent ^{1}H NMR spectra of cyanostilbenes **1** in $CDCl_3$ (300 MHz, 298 K) showing the aromatic, the amide and the methylene linked to the nitrogen atom and to the aromatic backbone protons.

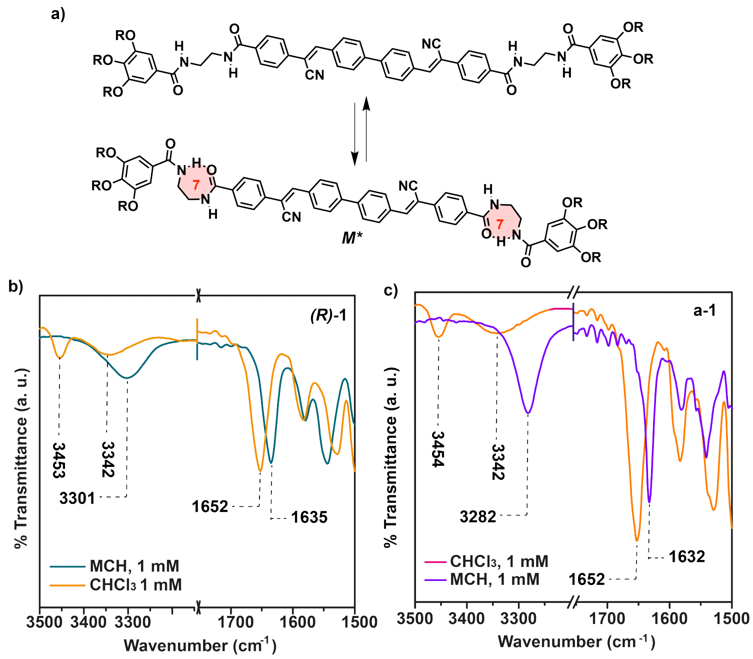

Figure S2. (a) Chemical equilibria of the free and metastable monomeric species ***M****** formed by the intramolecular H-bonding interactions between the NH and CO of the benzamide units. (b, c) Partial FTIR spectra of ***(R)*-1** (b) and **a-1** (c) in MCH and $CHCl_3$. The dotted lines show the wavenumber values for the NH and Amide I stretching bands.

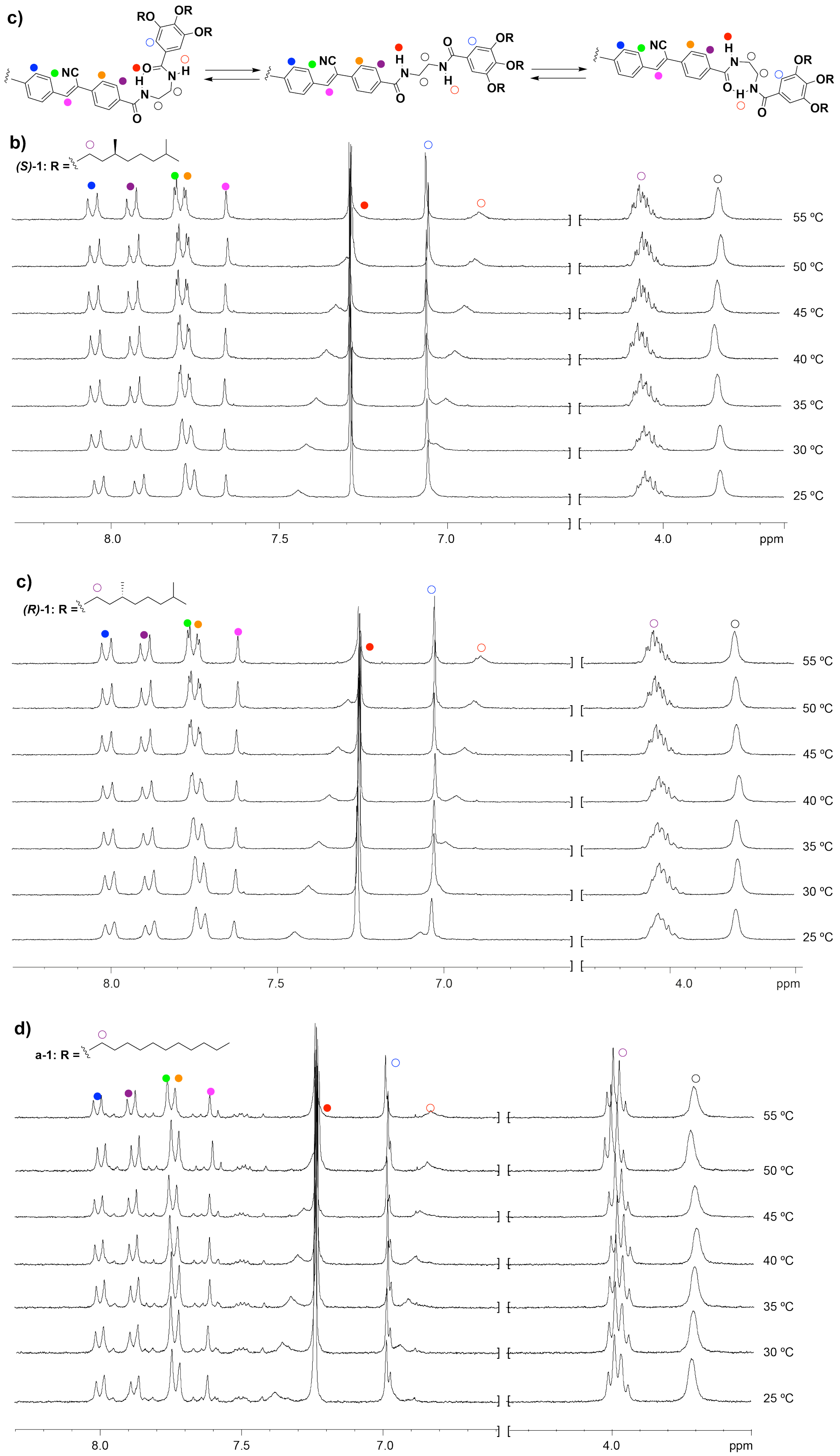

Figure S3. (a) Schematic illustration of the formation of the intramolecularly H-bonded, seven-membered pseudocycles. (b-d) VT-^{1}H NMR spectra of cyanostilbenes ***(S)*-1** (b), ***(R)*-1** (c) and **a-1** (d) in $CDCl_3$ (300 MHz, c_T = 1 mM) showing the aromatic, the amide and the methylene linked to the nitrogen atom and to the aromatic backbone protons.

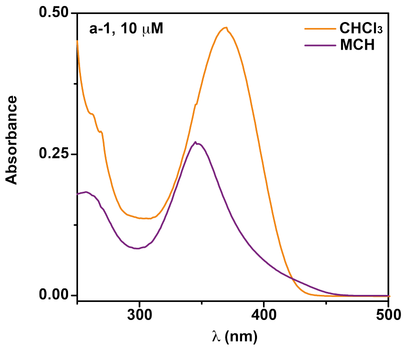

Figure S4. UV-Vis spectra of **a-1** in MCH and $CHCl_3$ at 20 °C at c_T = 10 μM.

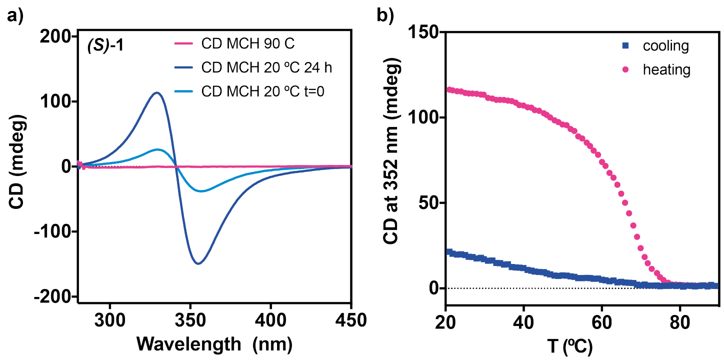

Figure S5. (a) CD spectra of ***(S)*-1** at different temperatures an at different times. (b) Variation of the dichroic response at λ = 352 nm observed in the cooling (blue dots) or heating (pink dots) process (MCH; c_T = 10 μM; cooling and heating rate: 1 ºC/min).

Table S1. g_{abs} and g_{lum} values for compounds ***(S)*-1** and ***(R)*-1** in MCH (c_T = 10 μM)

Compound	g_{abs} (355 nm)	g_{abs} (322 nm)	g_{lum}
***(S)*-1**	-0,012	0,007	0,009
***(R)*-1**	0,014	-0,008	-0,009

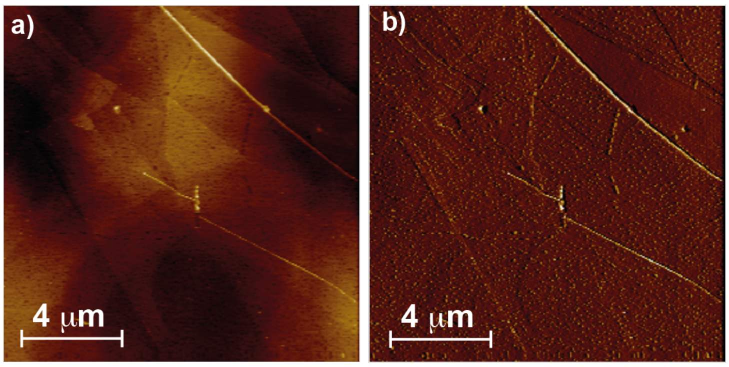

Figure S6. Height (a) and phase (b) AFM images of the rope-like fibrillar aggregates formed by ***(S)*-1** onto HOPG as surface. Experimental conditions: MCH; c_T = 10 μM, 20 °C.

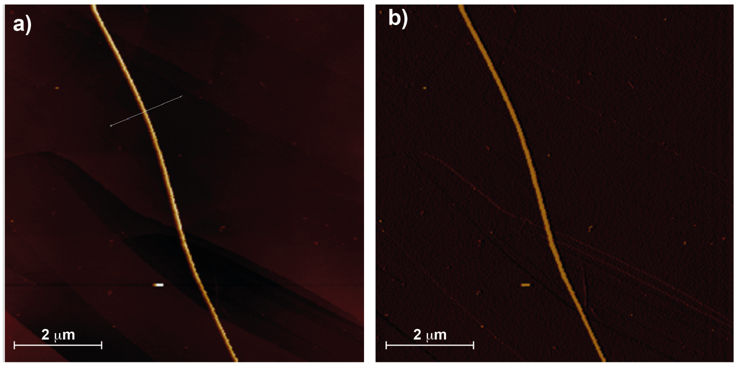

Figure S7. (Height (a) and phase (b) AFM images of the rope-like fibrillar aggregates formed by **a-1** onto HOPG as surface. Experimental conditions: MCH; c_T = 10 μM, 20 °C.

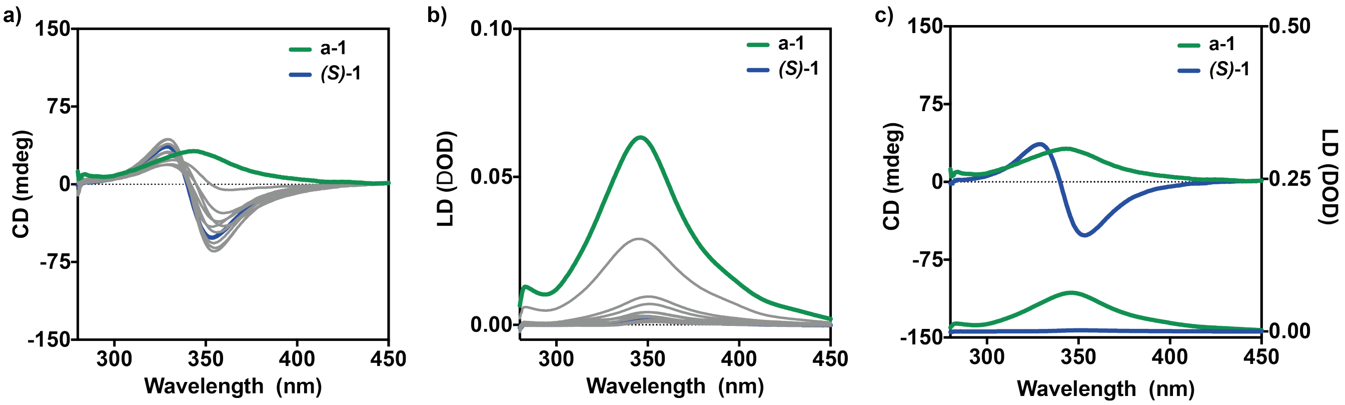

Figure S8. CD (a) and LD (b) spectra registered for the *SaS* experiment in which MCH solutions of **a-1** and ***(S)*-1** at c_T = 10 μM are mixed together at different ratio. (c) CD and LD spectra of pristine **a-1** and ***(S)*-1** in MCH at c_T = 10 μM.

3. Experimental section

General. All solvents were dried according to standard procedures. Reagents were used as purchased. All air-sensitive reactions were carried out under argon atmosphere. NMR spectra were recorded on a Bruker Avance 300 (^{1}H: 300 MHz; ^{13}C: 75 MHz), spectrometer at 298 K using partially deuterated solvents as internal standards. Coupling constants (*J*) are denoted in Hz and chemical shifts (δ) in ppm. Multiplicities are denoted as follows: s = singlet, d = doublet, t = triplet, m = multiplet, br = broad. Structural assignments were made with additional information from gHMQC experiments. FT-IR spectra were recorded on a Bruker Tensor 27 (ATR device) spectrometer. High resolution mass spectra (HRMS) were recorded on a FTMS Bruker APEX Q IV spectrometer. UV-Vis spectra were registered on a Jasco-V630 spectrophotometer equipped with a Peltier thermoelectric temperature controller. The spectra were recorded in the continuous mode between 220 and 450 nm, with a wavelength increment of 1 nm, a response time of 4 s, and a bandwidth of 1 nm. A 1 cm path length quartz cuvette (Hellma) was used. Thermal experiments were performed at constant heating rates of 1 K min^{-1} in methylcyclohexane. Circular dichroism (CD) measurements were performed on a Jasco-1500 dichrograph equipped with a Peltier thermoelectric temperature controller. The spectra were recorded in the continuous mode between 220 and 450 nm, with a wavelength increment of 1 nm, a response time of 4 s, and a bandwidth of 1 nm. A 1 cm path length quartz cuvette (Hellma) was used. The spectra were recorded in the continuous mode between 350 and 600 nm, with a wavelength increment of 1 nm, a response time of 4 s, and a bandwidth of 1 nm. A 1 cm path length quartz cuvette (Hellma) was used. A 1 cm path length quartz cuvette (Hellma) was used. Atomic Force Microscopy was performed on a SPM Nanoscope IIIa multimode microscope working on tapping mode with a RTESPA tip (Veeco) at a working frequency of ~235 kHz. High-resolution mass spectra (HRMS) were recorded on a MALDI Bruker daltonics Ultraflex TOF/TOF spectrometer.

Solvent-denaturation model for the supramolecular homopolymerization of luminogen ***(S)*-1**. The Gibbs free energy and the degree of cooperativity of the homopolymerization of chiral ***(S)*-1**have been extracted by applying the SD model in which aliquots of solutions of this luminogen in the good solvent $CHCl_3$ and the bad solvent MCH, keeping constant the total concentration. Fitting the variation of the absorbance versus the molar fraction of $CHCl_3$ and by using the equation $\Delta G' = \Delta G + mX$, being *m* a parameter that indicates the influence of the good solvent in the energetics of the process and *X* the molar fraction of the good solvent. For a detailed description of the derivation of the utilized equation, please, see: Korevaar, P. A.; Schaefer, C.; De Greef, T. F. A.; Meijer, E. W. Controlling Chemical Self-Assembly by Solvent-Dependent Dynamics. *J. Am. Chem. Soc.* **2012**, *134*, 13482)

4. Synthetic details and characterization

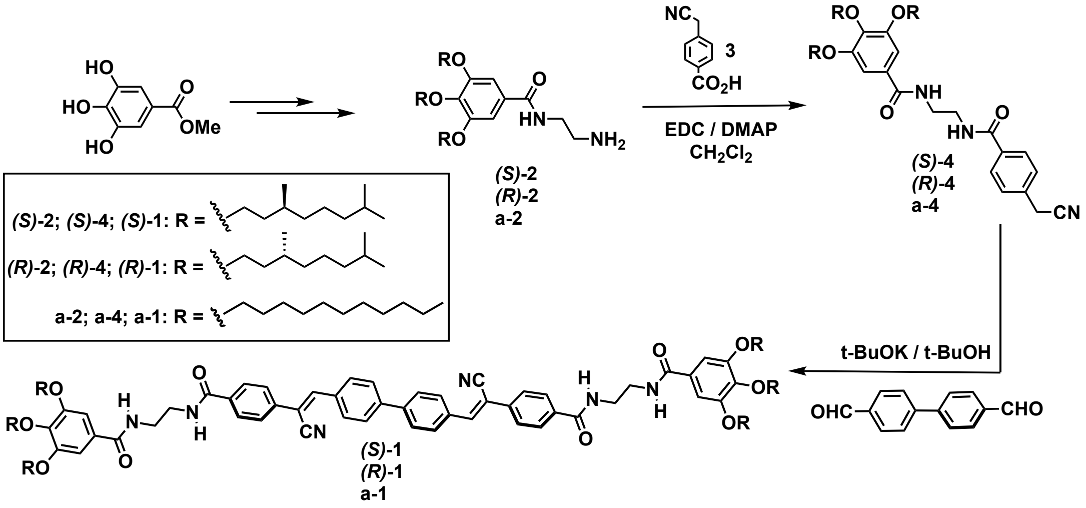

Scheme S1. Synthesis of the biphenyl cyanostilbenes **1**.

Compounds ***(S)*-2; *(R)*-2**, **a-2**; **3**, ***(S)*-4**; ***(R)*-4, and a-4** were prepared according to previously reported synthetic procedures and showed identical spectroscopic properties than those reported therein.[S1,S2]

[S1] López-Gandul, L.; Naranjo, C.; Sánchez, C.; Rodríguez, R.; Gómez, R.; Crassous, J.; Sánchez, L. Stereomutation and chiroptical bias in the kinetically controlled supramo-lecular polymerization of cyano-luminogens. Chem. Sci., 2022, 13, 11577.
[S2] Aparicio, F.; Cherumukkil, S.; Ajayaghosh, A. Sánchez, L. Color-Tunable Cyano-Substituted Divinylene Arene Luminogens as Fluorescent π-Gelators. Langmuir, 2016, 32, 284.

Synthesis and characterization of biphenyl cyanostilbenes. General procedure. To a solution of the corresponding benzamide **4** (1.7 mmol, 2.2 eq.) and 4,4'-biphenyl dicarbaldehyde (0.79 mmol, 1 eq.) in *t*-BuOH (4 mL/mmol of 4,4'-biphenyl dicarbaldehyde), 0.01 eq. of *t*-BuOK are added and the mixture is stirred at 50 ºC in an oil bath for 30 min in the absence of light. After this time, the resulting solid is filtered and washed with cold MeOH. Finally, it is dried under vacuum to obtain compounds **1** as pale, yellow solids.

N,N'-(((4,4'-((1Z,1'Z)-[1,1'-biphenyl]-4,4'-diylbis(1-cyanoethene-2,1-diyl))bis(benzoyl))bis(azanediyl))bis(ethane-2,1-diyl))bis(3,4,5-tris(((S)-3,7-dimethyloctyl)oxy)benzamide) (S)-1

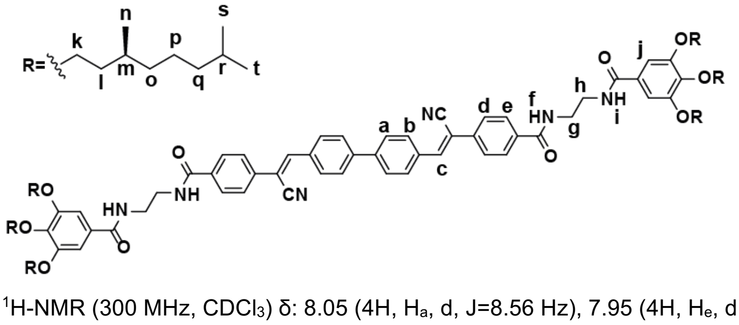

Yield: 112mg (78%). ^{1}H-NMR (300 MHz, CDCl$_3$) δ: 8.05 (4H, H_a, d, J=8.56 Hz), 7.95 (4H, H_e, d, J=8.02 Hz), 7.82 (4H, H_b, s), 7.70 (4H, H_d, s), 7.59 (2H, H_c, s), 7.56 (2H, H_f, br), 7.16 (2H, H_i, br), 7.05 (4H, H_j, s), 4.03 (12H, H_k, m), 3.72 (8H, H_{g+h}, br), 1.85 (6H, H_m, m), 1.68 (6H, H_r, br), 1.51 (12H, H_l, m), 1.38-1.08 (36H, H_{o-p}, br), 0.91 (18H, H_n, m), 0.85 (36H, H_t, d, J=5.3 Hz). ^{13}C-NMR (175 MHz, CDCl$_3$) δ: 169.0, 167.6, 153.2, 142.6, 142.0, 141.2, 137.3, 134.4, 133.0, 130.2, 128.7, 127.9, 127.5, 126.1, 117.7, 110.7, 105.5, 71.7, 67.5, 41.5, 40.8, 39.4, 39.3, 37.5, 37.4, 37.3, 36.4, 29.8, 29.7, 29.6, 28.0, 24.8, 24.7, 22.7, 22.6, 19.6. FT-IR (cm^{-1}): 766, 851, 1114, 1229, 1335, 1365, 1381, 1426, 1465, 1497, 1543, 1580, 1638, 2218, 2869, 2925, 2953, 3308. HRMS (MALDI-TOF) m/z: [M + H]+ Calcd for $C_{110}H_{161}N_6O_{10}$ 1725.2196; Found 1725.2188

N,N'-(((4,4'-((1Z,1'Z)-[1,1'-biphenyl]-4,4'-diylbis(1-cyanoethene-2,1-diyl))bis(benzoyl))bis(azanediyl))bis(ethane-2,1-diyl))bis(3,4,5-tris(((R)-3,7-dimethyloctyl)oxy)benzamide) *(R)*-1

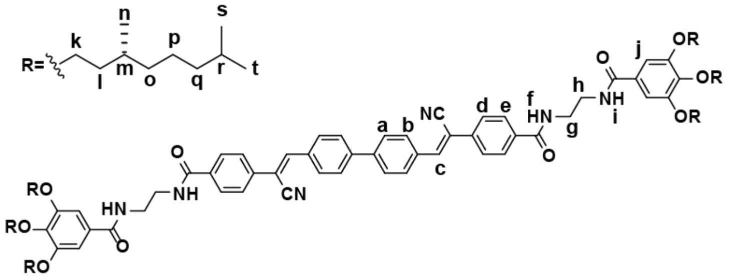

Yield: 126 mg (87%). ^{1}H-NMR (300 MHz, CDCl$_3$) δ: 8.00 (4H, H_a, d, J=8.56 Hz), 7.88 (4H, H_e, d, J=8.02 Hz), 7.74 (4H, H_d, s), 7.71 (4H, H_b, s), 7.63 (2H, H_c, s), 7.45 (2H, H_f, br), 7.08 (2H, H_i, br), 7.04 (4H, H_j, s), 4.04 (12H, H_k, m), 3.73 (8H, H_{g+h}, br), 1.86 (6H, H_m, m), 1.70 (6H, H_r, br), 1.52 (12H, H_l, m), 1.38-1.08 (36H, H_{o-p}, br), 0.92 (18H, H_n, m), 0.85 (36H, H_t, d, J=5.3 Hz). ^{13}C-NMR (75 MHz, CDCl$_3$) δ: 168.9, 167.9, 153.3, 142.5, 141.7, 141.2, 136.9, 134.5, 133.0, 130.3, 128.8, 128.1, 127.3, 125.9, 117.7, 110.4, 105.7, 71.9, 67.6, 40.7. 39.5, 39.4, 37.7, 37.5, 36.5, 31.4, 29.9, 29.8, 28.1, 24.9, 24.8, 22.8, 22.7, 19.7. FT-IR (cm^{-1}): 768, 853, 1109, 1231, 1332, 1366, 1379, 1425, 1466, 1498, 1542, 1581, 1638, 2218, 2870, 2924, 2952, 3308. HRMS (MALDI-TOF) m/z: [M + H]+ Calcd for $C_{110}H_{161}N_6O_{10}$ 1725.2196; Found 1725.2202

N,N'-(((4,4'-((1Z,1'Z)-[1,1'-biphenyl]-4,4'-diylbis(1-cyanoethene-2,1-diyl))bis(benzoyl))bis(azanediyl))bis(ethane-2,1-diyl))bis(3,4,5-tris(dodecyloxy)benzamide) a-1

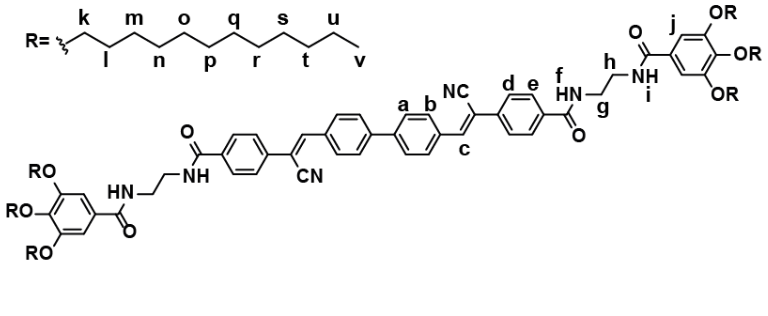

Yield: 116mg (77%). ^{1}H-NMR (300 MHz, $CDCl_3$) δ: 7.88 (4H, H_a, d, J=8.56 Hz), 7.77 (4H, H_e, d, J=8.02 Hz), 7.61 (4H, H_d, s), 7.58 (4H, H_b, s), 7.54 (2H, H_c, s), 7.22 (2H, H_f, br), 7.04 (4H, H_j, s), 6.87 (2H, H_i, br), 4.03 (12H, H_k, m), 3.76 (8H, H_{g+h}, br), 1.79 (12H, H_l, m), 1.49-0.88 (120H, H_{m-v}, m). ^{13}C-NMR (175 MHz, $CDCl_3$) δ: 169.0, 167.5, 153.1, 142.6, 134.4, 130.2, 128.6, 127.9, 127.6, 126.1, 105.5, 73.5, 69.3, 31.9, 30.3, 29.78, 29.76, 29.74, 29.72, 29.70, 29.62, 29.46, 29.42, 29.40, 29.38, 26.14, 26.10, 22.7, 14.2. FT-IR (cm^{-1}): 700, 755, 1116, 1232, 1334, 1365, 1461, 1494, 1528, 1583, 1652, 2216, 2855, 2927, 2959, 3020. HRMS (MALDI-TOF) m/z: [M + H]+ Calcd for $C_{122}H_{185}N_6O_{10}$ 1894.8460; Found 1894.8465

Collection of NMR spectra

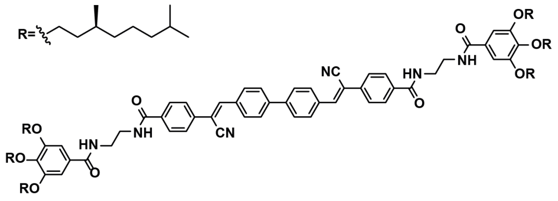

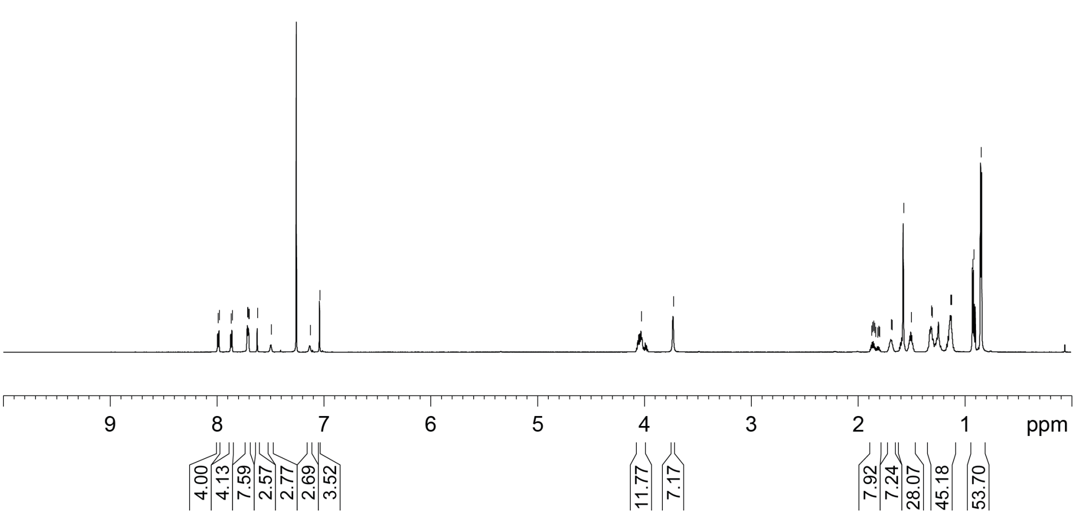


```
Current Data Parameters
NAME          Q05FG53
EXPNO         1
PROCNO        1

F2 - Acquisition Parameters
Date_         20250404
Time          h    12.29
INSTRUM       spect
PROBHD        Z75813_0001 (C
PULPROG       zg30
TD            32768
SOLVENT       3  CDCl
NS            2
DS            2
SWH           10504.202 Hz
FIDRES        0.641126 Hz
AQ            1.5597568 sec
RG            6.35
DW            47.600 usec
DE            6.50 usec
TE            K    300.0
D1            1.00000000 sec
TD0           1
SFO1          700.1742010 MH
NUC1          1H
P0            2.50 usec
P1            7.50 usec
PLW1          10.00000000

F2 - Processing parameters
SI            32768
SF            700.1700217 MH
WDW           EM
SSB           0
LB            0.30 Hz
GB            0
PC            1.00
```

^{1}H NMR spectrum of compound ***(S)*-1** (300 MHz, $CDCl_3$, 298 K).

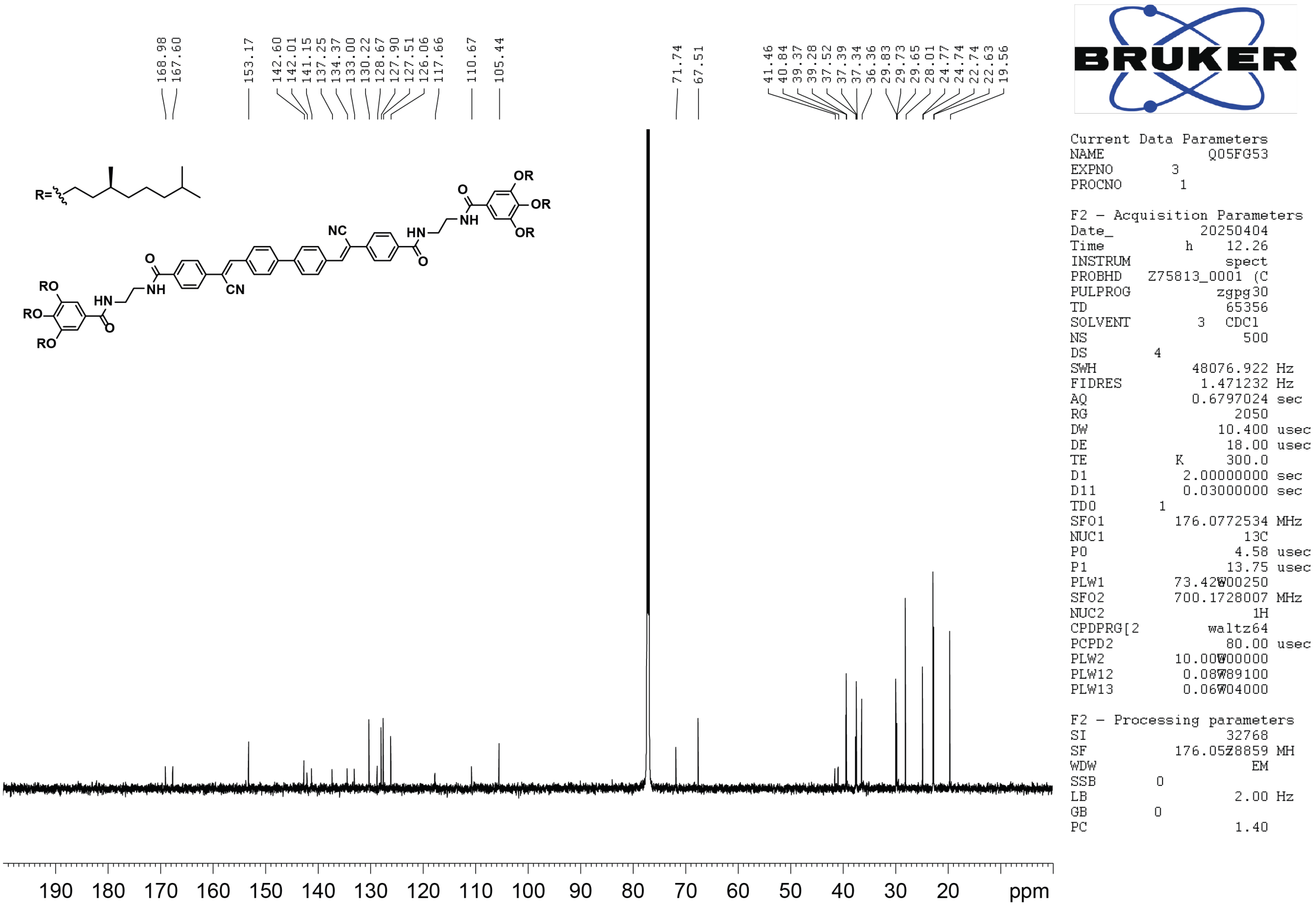

^{13}C NMR spectrum of compound ***(S)*-1** (175 MHz, $CDCl_3$, 298 K).

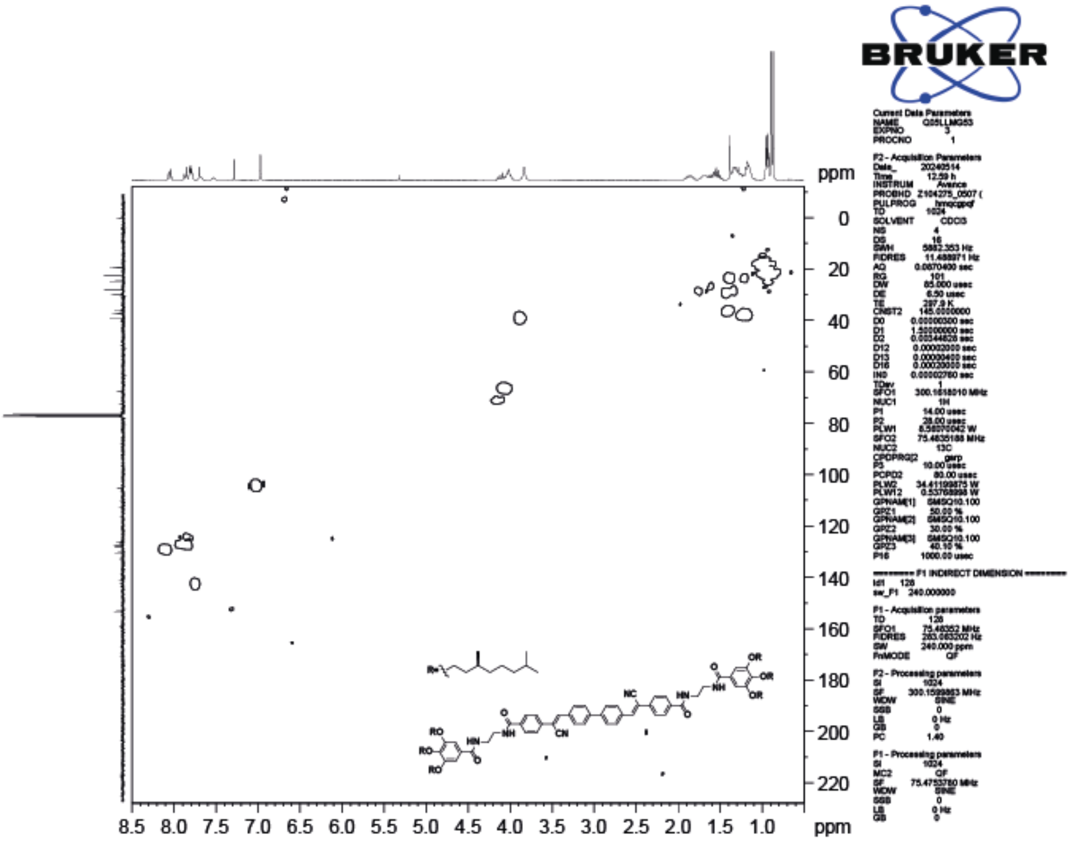

^1H-^{13}C HMQC spectrum of compound ***(S)*-1** ($CDCl_3$, 298 K).

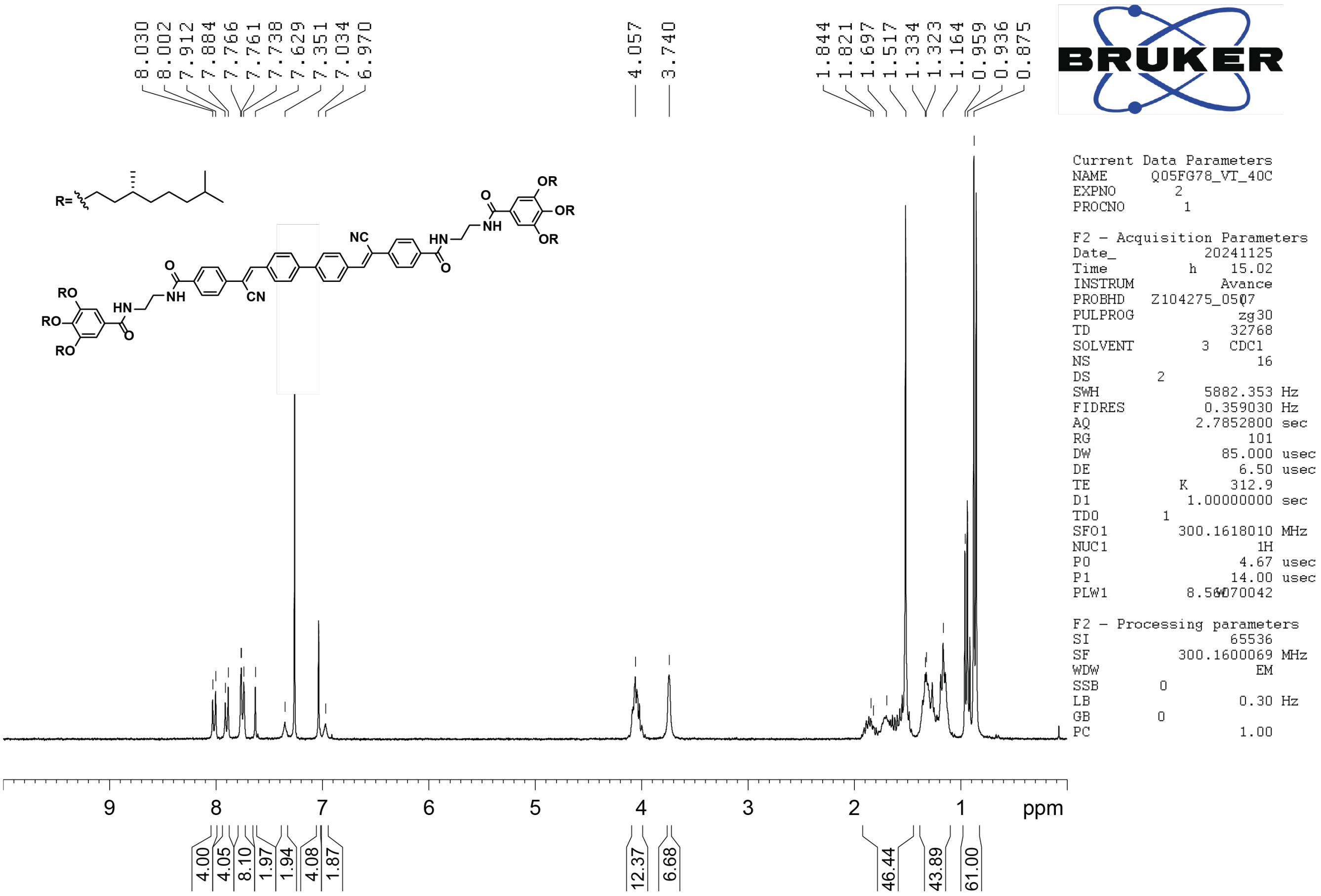

^{1}H NMR spectrum of compound ***(R)*-1** (300 MHz, $CDCl_3$, 298 K).

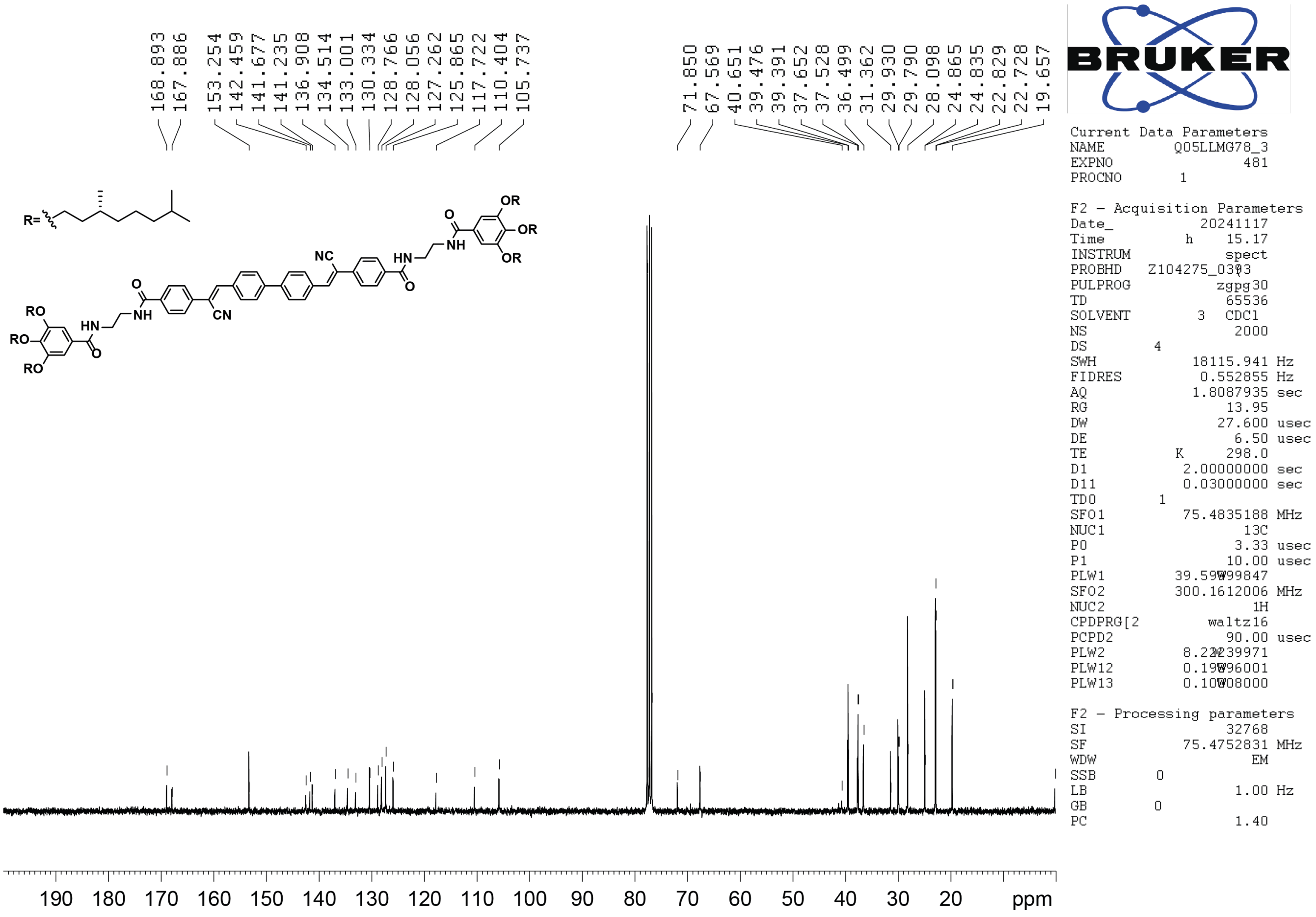

^{13}C NMR spectrum of compound ***(R)*-1** (75 MHz, $CDCl_3$, 298 K).

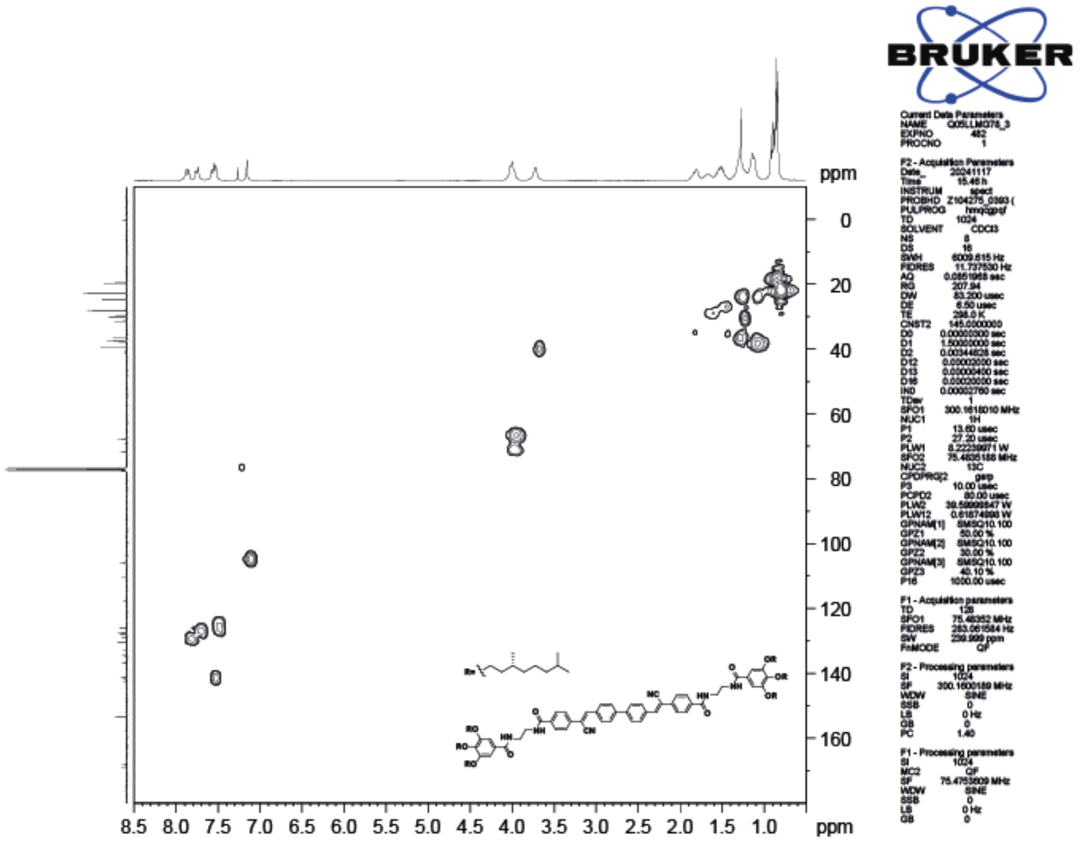

^{1}H-^{13}C HMQC spectrum of compound ***(R)*-1** ($CDCl_3$, 298 K).

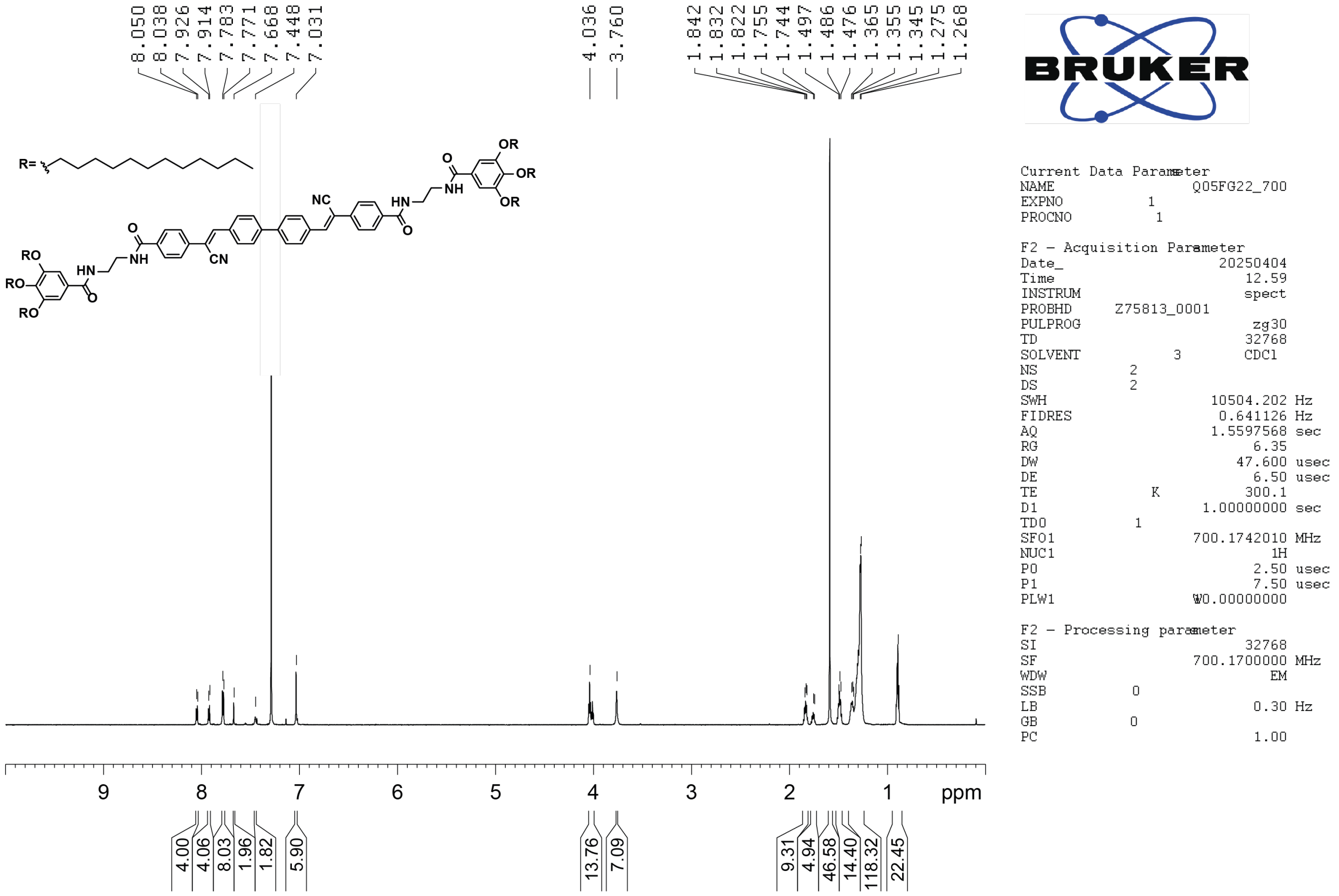

^{1}H NMR spectrum of compound **a-1** (300 MHz, $CDCl_3$, 298 K).

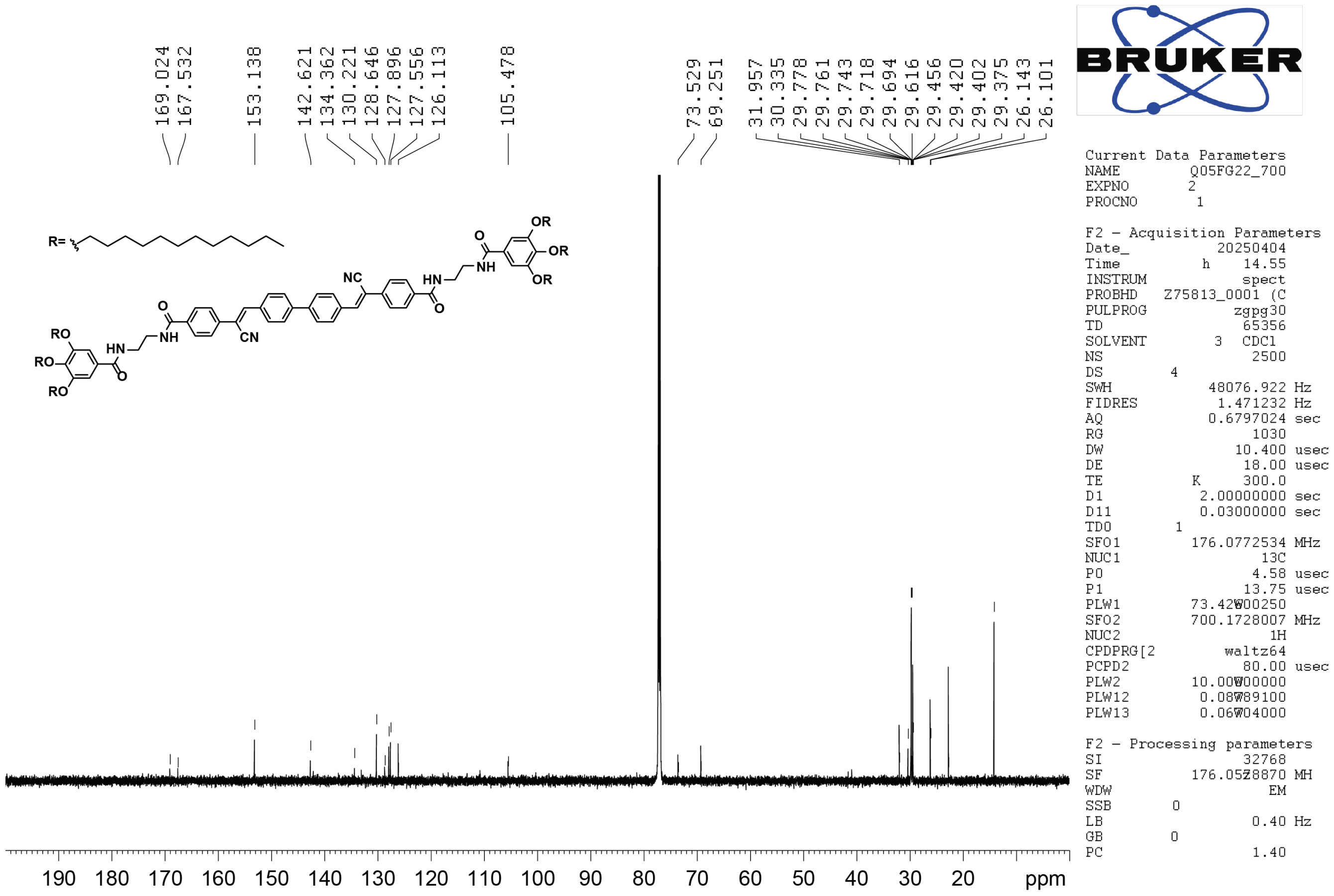

^{13}C NMR spectrum of compound **a-1** (175 MHz, $CDCl_3$, 298 K).

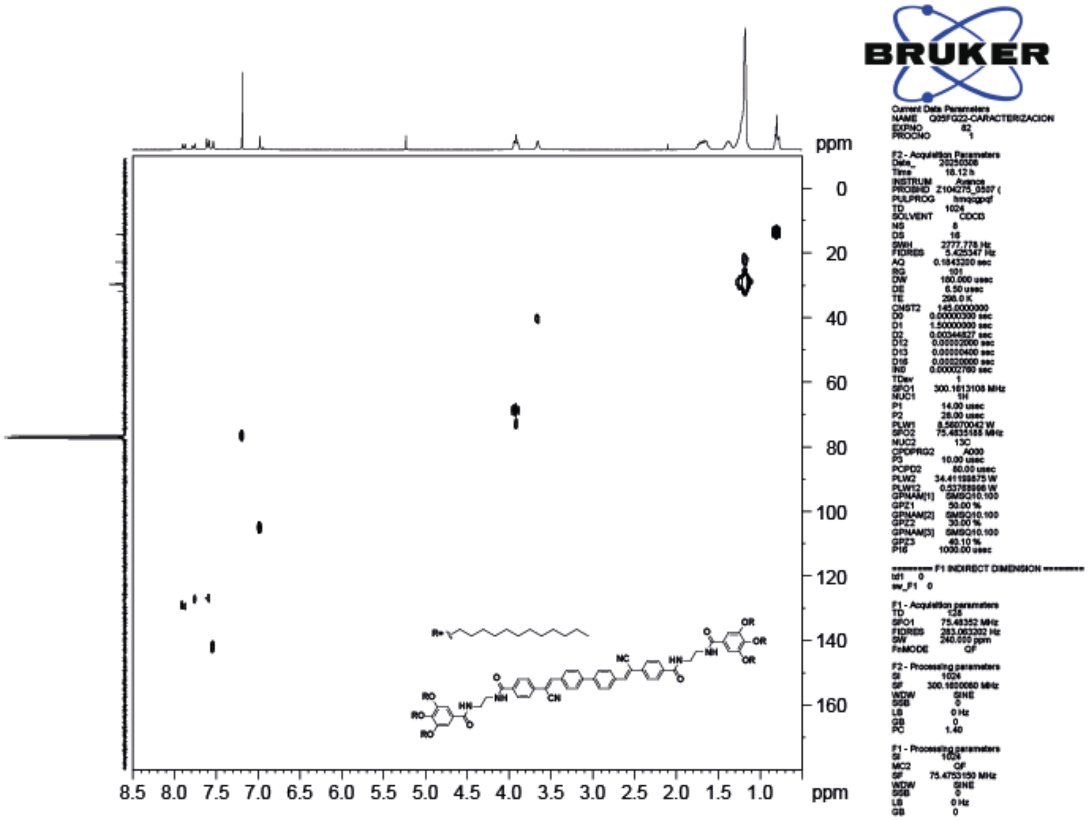

^{1}H-^{13}C HMQC spectrum of compound **a-1** ($CDCl_3$, 298 K).
